# Spatio-Temporal Expression and Functional Involvement of Transient Receptor Potential Vanilloid 1 in Diabetic Mechanical Allodynia in Rats

**DOI:** 10.1371/journal.pone.0102052

**Published:** 2014-07-14

**Authors:** Yuan-Yuan Cui, Hao Xu, Huang-Hui Wu, Jian Qi, Juan Shi, Yun-Qing Li

**Affiliations:** 1 Department of Anatomy, Histology and Embryology & K.K. Leung Brain Research Centre, Preclinical School of Medicine, Fourth Military Medical University, Xi'an, China; 2 Institution of Basic Medical Science and Lab of Cell Biology & Translational Medicine, Xi'an Medical University, Xi'an, China; 3 Department of Anesthesiology, Fuzhou General Hospital Affiliated to Fujian Medical University, Fuzhou, China; Beijing Institute of Radiation Medicine, China

## Abstract

Diabetic neuropathic pain (DNP) is one of the most common clinical manifestations of diabetes mellitus (DM), which is characterized by prominent mechanical allodynia (DMA). However, the molecular mechanism underlying it has not fully been elucidated. In this study, we examined the spatio-temporal expression of a major nociceptive channel protein transient receptor potential vanilloid 1 (TRPV1) and analyzed its functional involvement by intrathecal (*i.t*.) application of TRPV1 antagonists in streptozocin (STZ)-induced DMA rat models. Western blot and immunofluorescent staining results showed that TRPV1 protein level was significantly increased in the soma of the dorsal root ganglion (DRG) neurons on 14 days after STZ treatment (DMA 14 d), whereas those in spinal cord and skin (mainly from the central and peripheral processes of DRG neurons) had already been enhanced on DMA 7 d to peak on DMA 14 d. qRT-PCR experiments confirmed that TRPV1 mRNA level was significantly up-regulated in the DRG on DMA 7 d, indicating a preceding translation of TRPV1 protein in the soma but preferential distribution of this protein to the processes under the DMA conditions. Cell counting assay based on double immunostaining suggested that increased TRPV1-immunoreactive neurons were likely to be small-sized and CGRP-ergic. Finally, single or multiple intrathecal applications of non-specific or specific TRPV1 antagonists, ruthenium red and capsazepine, at varying doses, effectively alleviated DMA, although the effect of the former was more prominent and long-lasting. These results collectively indicate that TRPV1 expression dynamically changes during the development of DMA and this protein may play important roles in mechanical nociception in DRG neurons, presumably through facilitating the release of CGRP.

## Introduction

Diabetic neuropathic pain (DNP) is one of the most common complications that affects approximately 20% of patients with diabetes mellitus (DM) in the world [1], [2]. The primary clinical symptoms of DNP are sensory disorders of distal limbs, such as spontaneous pain, hyperalgesia and allodynia which might be caused by peripheral demyelination, degeneration of myelinated sensory fibers and impaired unmyelinated C-fibers [3], [4]. DNP reduces the quality of lives of the patients suffering from DM. However, DNP is an extremely intractable illness, for which the most of treatments are found only partially effective. Lack of exact understanding about how DNP develops could potentially hinder the development of its effective therapy.

The transient receptor potential vanilloid 1 (TRPV1) is a ligand-gated non-selective cation channel that is highly expressed in the central and peripheral nervous systems [5], [6]. Acting as a polymodal signal transducer for thermal, proton and chemical stimuli, this channel plays key roles in potentially injurious events occurring in the neurons [7]_ENREF_5. Evidence from rodent models suggested that, under inflammatory and neuropathic conditions, the expression level and/or the functionality of TRPV1 were up-regulated in dorsal root ganglion (DRG), spinal dorsal horn and the endogenous antinociceptive center, periaqueductal grey [8]–[10]. These findings imply the essential involvement of TRPV1 channel in pain signal transduction and integration. Consistent with this view, thermal hyperalgesia and mechanical allodynia derived from inflammation or nerve injury were reported to be alleviated by down-regulating TRPV1 expression or pharmacologically inhibiting the channel activity [11]–[13].

Streptozocin (STZ) is a commonly used agent to induce type 1 diabetes in rodent DNP models. Similar to the clinical complaints of patients, the symptoms of DNP in rodent STZ-induced DM models were manifested as mechanical allodynia and thermal hyperalgesia [2], [14], where expression of TRPV1 in DRG of the latter condition was elevated [15], [16]. Mechano-sensation/transduction is the conserved ability for extant organisms ranging from bacteria to mammals. Strong mechanical hypersensitivity severely affects the daily lives of DM patients; their dressing behaviors are greatly disturbed by avoidance of frequent ‘painful’ contact with clothes. Despite this clinical importance, the pathogenic mechanisms underlying the mechanical hypersensitivity of DM patients remain large unclear. TRPV1 was regarded as an essential molecular mediator of thermal hyperalgesia in various acute and chronic pain models [17], but different lines of evidence have recently shown that this protein may also contribute to the mechanical sensitivity of the nervous system [18], [19]. For instance, a shorter splice variant of TRPV1 lacking N-terminus which is specifically expressed in the hypothalamic circumventricular organs, may serve to transduce hypertonic stimuli into electrical signals thereby modulating the release of vasopressin; the process is essential to regulate the body fluid balance. Furthermore, the afferent discharges generated by urinary bladder distention were found to be significantly attenuated by the antagonists for TRPV1 or its genetic deletion [20]. DM is characterized by high blood glucose concentration and high osmolality [21]. Thus, elucidating TRPV1 expression pattern and functionality in different DMA states may be informative to elucidate nociceptive signaling in peripheral sensory neurons.

In this study, we investigated the spatio-temporal expression profile of TRPV1 in primary sensory neurons and their afferent terminals, and the effects of TRPV1 antagonists, ruthenium red (RR) and capsazepine (CPZ) under the conditions of DMA. We aimed to clarify the relationship between TRPV1 expression and its native function in mechanically hypersensitized rats *in vivo*.

## Materials and Methods

### Animal manipulations

Adult male *Sprague-Dawley* rats (180–220 g) were housed in plastic cages (two in each cage), and maintained on a 12 h light-dark cycle (lights on at 8:00 and off at 20:00) at ambient temperature (20
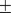
2°C) with abundant food and water supply. All rats were used for experiments within at least 7 days after arrival and each rat was used only once. Animal experiments were performed according to the ethical guidelines of the International Association for the Study of Pain [22] after the approval from the Animal Use and Care Committee for Research and Education of the Fourth Military Medical University (Xi'an, China).

### DM model generation

STZ is one of the most prominent diabetogenic chemical components in experimental diabetes research [2]. Rats were randomized to receive either STZ (Sigma-Aldrich, St. Louis, MO, USA) or vehicle treatment. Those which received vehicles alone were used as vehicle control. DM model was generated by intraperitoneal (*i.p*.) administration of STZ (2%, 60 mg/kg) that had been freshly dissolved in vehicle (citric acid:sodium citrate  = 1∶1.32, pH 4.5). Since the increase of blood glucose concentration is regarded as the key index for the successful induction of DM animal model, the glucose level of blood samples from the tail vein on DM 3 d was measured with a glucometer (Accu-Chek Active, Roche, Basel, Switzerland). Only those with the non-fasting blood glucose concentration higher than 16.7 mmol/L (300 mg/dl) were regarded as DM rats [2] and used for experiments. The body weight was also used as another indicator for diabetic state.

### Behavioral analysis and DMA model identification

To minimize environmental influences and individual variability, all animals were acclimated to the experimental environment for at least 30 min before starting behavioral tests. Rats were put in Plexiglas boxes (30 cm×30 cm×50 cm) placed on an elevated metal mesh floor. The von Frey filaments (0.6, 1, 1.4, 2, 4, 6, 8, 10, and 15 g, respectively; Stoelting, Kiel, WI, USA) were pressed on the plantar surface for approximately 4–5 s, which was repeated 5 times at a 5-min interval on each hind paw. The minimal value that caused at least 3 responses was considered as the paw withdrawal threshold (PWT). PWT tests were firstly performed 3 days before STZ injection to get baseline, and repeated 0, 7, 14, 21 and 28 days after STZ administration to estimate DMA-related behaviors. All behavioral experiments were performed in a blind manner between 9:00 am and 6:00 pm. The rats with decreased PWT at 7 and/or 14 days after STZ treatment were used for further experiments [23]. Paw withdrawal response to noxious thermal stimuli (detected by the Hargreaves method) [24] was tested according to our previously published protocol [25].

### Western blot

Rats were anesthetized with sodium pentobarbital (65 mg/kg, *i.p*.), and the L_4-5_ segments of SDH and bilateral DRGs were immediately removed and subjected to Western blot analysis (n = 6 for each group). After measuring the protein concentration of each sample, the equal amounts of protein (30 µg) from SDHs or DRGs were denatured at 100°C for 5 min and electrophoresed on an 8% SDS-PAGE. The proteins were transferred onto polyvinylidene fluoride membrane (PVDF, ? 0.45 µm, Millipore, Billerica, MA, USA). The membranes were blocked by Tris-buffered saline supplemented with 0.02% Tween (TBS-T) and 5% non-fat dry milk for 1 h at room temperature (RT), and incubated overnight at 4°C with rabbit anti-TRPV1 antibody (1∶1000; SAB3501027; Sigma-Aldrich) and mouse anti-β-actin antibody (1∶5000; A2228; Sigma-Aldrich), respectively. Then horseradish peroxidase-conjugated anti-rabbit (1∶5000; AP106P; Millipore) or anti-mouse secondary antibodies (1∶5000; AP124P; Millipore) were used to incubate the membranes for 1 h at RT. Between respective steps, the immunoblots were rinsed with TBS-T 3 times for 10 min. All protein bands were visualized with the ECL kit (Amersham Pharmacia Biotech, Piscataway, NJ, USA) and then detected by using the ChemiDoc Imaging System (Bio-Rad, Berkeley, CA, USA). The density of the bands were quantified after background subtraction and then normalized with respect to the β-actin level.

### Quantitative real-time PCR (qRT-PCR)

Rats were killed after sodium pentobarbital anesthesia (65 mg/kg, *i.p*.) and bilateral L_4–5_ DRGs were quickly harvested on ice and transferred to a deep freezer (−80°C) for later use (n = 4–6 for each group). Total RNA was extracted using the Trizol reagent (GIBCO BRL, Grand Island, NY, USA) and the equal amounts of RNA were reversely transcribed into cDNAs with the Prime Script RT reagent Kit (Takara, Kyoto, Japan) according to the manufacturer's protocol. TRPV1 and GAPDH mRNA levels were assayed by the Mini-Opticon real-time PCR detection system (Bio-Rad, Hercules, CA, USA) with SYBR Green master mix reagent (Takara, Otsu, Japan) according to the manufacturer's protocol. All data were analyzed by using the Opticon Monitor software (version 3.1; Bio-Rad). The primer sequences used for qRT-PCR were as follows: TRPV1 (NM_031982.1), 5′-tgactgacagcgagttca-3′ (forward) and 5′-tgtgtagctggcattgac-3′ (reverse); GAPDH (NM_017008.3), 5′-gccactcagaagactgtgga-3′ (forward) and 5′-gttcagctctgggatgacct- 3′ (reverse).

### Immunohistochemistry

Rats were deeply anaesthetized and perfused transcardially with 100 ml of 0.9% normal saline, followed by 500 ml of 0.1 mol/L phosphate buffer (PB, pH 7.2) containing 4% (w/v) paraformaldehyde. Subsequently, the L_4–5_ segments of spinal cord (SC), the corresponding DRGs and plantar skin were removed, postfixed in the same fixative for 2–4 hours and cryoprotected overnight at 4°C in 0.1 mol/L PB containing 30% (w/v) sucrose. Transverse sections (30 µm in thickness for SC, 10 µm for skin) and horizontal sections (10 µm in thickness for DRG) were cut in a cryostat (CM1800; Leica, Heidelberg, Germany) and collected into 6 dishes or slides. Each dish or slide included a complete set of serial sections. The first set of sections was used for immunofluorescent staining of TRPV1. Briefly, the sections in the first dish or slide were rinsed in 0.01 mol/L phosphate-buffered saline (PBS, pH 7.2) and blocked with 10% fetal bovine serum in 0.01 mol/L PBS for 1 h at RT. The sections were then sequentially incubated with: (1) rabbit anti-TRPV1 antibody (1∶1000; SAB3501027; Sigma-Aldrich) at 4°C for 48 h; (2) biotinylated donkey anti-rabbit IgG (1∶500; AP182F; Millipore) for 6–8 h at RT; (3) Alexa Fluor 594-labeled avidin D (1∶1000; S32356; Invitrogen; only for skin) or fluorescein isothiocyanate (FITC)-labeled avidin D (1∶1000; A-2001; Vector, Burlingame, CA, USA) together with DAPI (4′,6-diamidino-2-phenylindole; 1∶1000; D9542; Sigma-Aldrich) for 1–2 h at RT. DAPI staining was employed to count the number of DRG neurons (pictures not shown). Between the steps, 0.01 mol/L PBS was used to thoroughly rinse the sections. The incubation medium used for the primary and secondary antibodies (including those for the following double-labeled staining) was 0.01 mol/L PBS (pH 7.4) which contained 2% normal donkey serum, 0.3% Triton X-100 (PBS-X, pH 7.4), 0.25% γ-carrageen, and 0.05% sodium azide (NaN_3_). The medium used for FITC-avidin was 0.01 mol/L PBS (pH 7.4) containing 0.3% Triton X-100.

Double-labeled immunofluorescent staining for TRPV1/neurofilaments 200 kDa (NF200) and TRPV1/calcitonin gene-related peptide (CGRP) were carried out with the second and third sets of sections, respectively. Briefly, the sections in the second or third series were incubated with a mixture of rabbit anti-TRPV1 (1∶1000; SAB3501027; Sigma-Aldrich) and mouse anti-NF200 antibody (1∶500; N0142; Sigma-Aldrich) or goat anti-CGRP antibody (1∶500; ab36001; Abcam, Cambridge, MA, USA) at 4°C for 48 h, respectively. After rinsing, sections were subsequently incubated with a mixture of Alexa Fluor 594-labeled donkey anti-rabbit IgG (1∶500; A21207; Invitrogen, Carlsbad, CA, USA) and Alexa Fluor 488-labeled donkey anti-mouse IgG (1∶500; A21202; Invitrogen) or Alexa Fluor 488-labeled donkey anti-goat IgG (1∶500; A11055; Invitrogen) for 6–8 h at RT. Double-immunofluorescent staining for TRPV1 and fluorescein labeled griffonia simplicifolia lectin I-isolectin B4 (IB4; 1∶500; FL-1201; Vector) were conducted with the fourth set of sections. In short, the sections were incubated with rabbit anti-TRPV1 antibody (1∶1000; SAB3501027; Sigma-Aldrich) at 4°C for 48 h and then with a mixture of IB4 (1∶500; FL-1201; Vector) and Alexa Fluor 594-labeled donkey anti-rabbit IgG (1∶500; A21207; Invitrogen) for 6–8 h at RT.

SC sections were mounted onto the clean glass slides pre-coated with gelatin. Together with those from the DRG and skin, all stained sections were cover-slipped with mounting medium (0.05 mol/L PBS containing 5% (v/v) glycerin and 2.5% (w/v) triethylenediamine). Images were observed by a confocal laser scanning microscope (FV1000, Olympus, Tokyo, Japan) and digital images were captured with the Fluoview 1000 software (Olympus). The specificity of staining was tested with the fifth and sixth sets of sections by omitting the primary specific antibodies, where no immunoreactive staining was detected (data not shown).

### Neural cell counting and image analysis

For quantitative analysis of the immunohistochemical results, we counted the numbers of single-labeled (TRPV1, NF200, CGRP, or IB4) and double-labeled (TRPV1/NF200, TRPV1/CGRP, or TRPV1/IB4) neurons, respectively. Only the neurons crossing nuclei were selected to count. The percentage of single-labeled neurons over total neurons and that of double-labeled over classical neuronal markers (NF200, CGRP or IB4)-IR neurons were calculated from 4–5 sections obtained from the L_4_ or L_5_ DRG. The cross-sectional area of TRPV1-positive neurons in DRGs, as well as optical density (OD) of TRPV1 immunoreactivity in DRGs, SDHs, epidermal and dermis (the area 0–50 µm below the dermo-epidermal junction, method see [26]) were measured using the free NIH software ‘Image J’v1.46r.

### Behavioral pharmacology

In order to estimate the contribution of TRPV1 to DMA and thermal hyperalgesia, rats were injected intrathecally with RR or CPZ. Under chloral hydrate anesthesia (7%, 350 mg/kg, *i.p*.), a 2–3 cm incision was made at the midline of the rat back at the L_4–5_ vertebrate level. A polyethylene tube (PE-10, ID 0.28 mm, OD 0.61 mm; No. 427401; BD, Sparks, USA) was passed into the subarachnoid space of the lumbar enlargement of SC (L_4–5_). The free end of the tube was exposed out of the rat's neck skin and fixed *in situ*. 10 µl of saline was infused into the tube before the exposed end was sealed. The rats were allowed to recover for 3–5 days and only those which acted without neurological abnormality and exhibited complete paralysis of bilateral hind legs shortly after injection of lidocaine (2%, 10 µl) were used for the following experiments. RR and CPZ (both from Sigma-Aldrich) of varying doses (RR, 0.1, 0.4 or 1.6 µg/kg; CPZ, 25, 100 or 400 µg/kg) in a volume of 10 µl were intrathecally applied to rats on the 14th day for single administration or on the 14–20th consecutive days for multiple administrations, respectively. To avoid the dead space volume problem, another 10 µl of saline was subsequently filled into the tube each time after drug administration. For single application, the paw withdrawal tests were performed at 2, 4, 6, 8 and 24 h after administrating either of the two drugs to evaluate their dynamic features. To evaluate the accumulative effects of drugs, we measured the PWT for multiply-treated animals once per every consecutive day. Control group received injection of the same amount of saline and underwent similar manipulations to experimental group.

### Data analysis

The data are expressed as mean
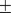
S.E.M. One-way analysis of variance (ANOVA) with repeated measurement followed by *Bonferroni*'*s* post hoc test was used for multiple comparisons. All these data were analyzed by using the GraphPad Prism version 5.01 for Windows (Graph Pad Software, San Diego, CA, USA, www.graphpad.com). *P*<0.05 was considered as statistical significance.

## Results

### Evaluation of hyperglycemia and mechanical allodynia in STZ-induced DM rats

**Figure 1:**
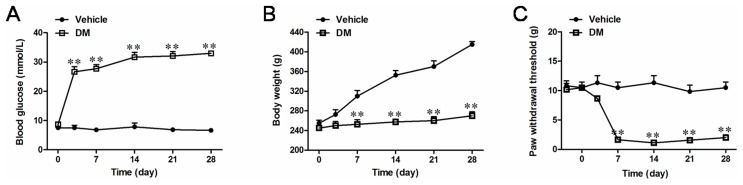
DM and DMA rat model induced by STZ injection. Time course of blood glucose concentration (**A**), body weight (**B**) and paw withdrawal threshold (**C**) alterations after STZ or citrate buffer (vehicle) treatment. Data are presented as mean
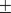
S.E.M. (n = 30), **P<0.01 *vs*. vehicle control.

In the present study, vehicle-treated rats maintained normal blood glucose level throughout the experimental period (7.5
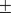
0.9 mmol/L, n = 30; Fig. 1A). In contrast, about 90% of rats developed hyperglycemia (26.7
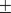
1.7 mmol/L, n = 30) from the third day after STZ injection, which was maintained throughout the following experimental days. Another important index to assess DM, body weight increase, was also significantly impaired after the injection of STZ ensuring the successful establishment of DM in the present rat model (Fig. 1B). Out of the DM rats established in this way, about 53% exhibited significantly decreased PWT on DMA 7 d, which continued until DMA 28 d. The incidence of DMA is comparable to that reported in previous report [23]. We thus defined these rats as DMA rats and used them for further experiments (Fig. 1C).

### Characteristics of the spatio-temporal expression of TRPV1

**Figure 2:**
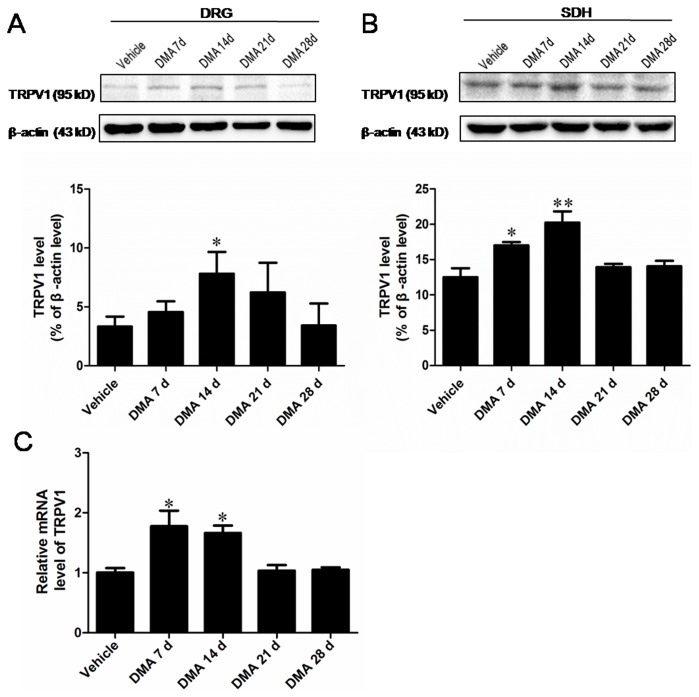
Expression tendency of TRPV1 in DRG and SDH with the progression of DMA. **A** and **B**, Western blot assay of TRPV1 in extracts of DRG (**A**) and SDH (**B**). Upper lane, typical blotting band. Lower lane, the densitometric analysis reveals the significant increase of TRPV1 protein in DRG on DMA 14 d and increase of TRPV1 in SDH on DMA 7 d and 14 d. **C**, Quantitative RT-PCR assay of TRPV1 expression at mRNA level revealing the marked increase of TRPV1 transcripts in DRG on DMA 7 d and 14 d. Data are represented as the mean
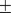
S.E.M (n = 6).*P<0.05, **P<0.01 *vs*. vehicle control.

In the next step, we evaluated the expression pattern of TRPV1 in the L_4-5_ DRGs and the corresponding segments of SDH with respect to DMA development. Western blot experiments showed that the expression level of TRPV1 protein in DRGs started to elevate on DMA 7 d and became significantly up-regulated on DMA 14 d (Fig. 2A; *P*<0.05 *vs*. vehicle). On the later phase of DMA (from 21 d to 28 d), however, the expression of TRPV1 declined, eventually returning to a level indistinguishable from that of vehicle. The qRT-PCR analysis demonstrated a similar transient increase in TRPV1 mRNA level; (Fig. 2C). The expression of TRPV1 protein in SDHs exhibited a slightly different profile as compared with DRGs. The up-regulation of TRPV1 protein was already significant on DMA 7 d, but completely abolished on DMA 21 d (Fig. 2B), suggesting earlier changes in the increase of TRPV1 expression in SDHs than in DRGs. In aggregate, the above findings indicate that the development of DMA is accompanied by the up-regulation of TRPV1 expression, which is transient and spatially different. Moreover, the coincidence of TRPV1 up-regulation and decreased PWT in the early phase (DMA 7 d to 14 d) may imply their causal relationship in the development of DMA.

**Figure 3:**
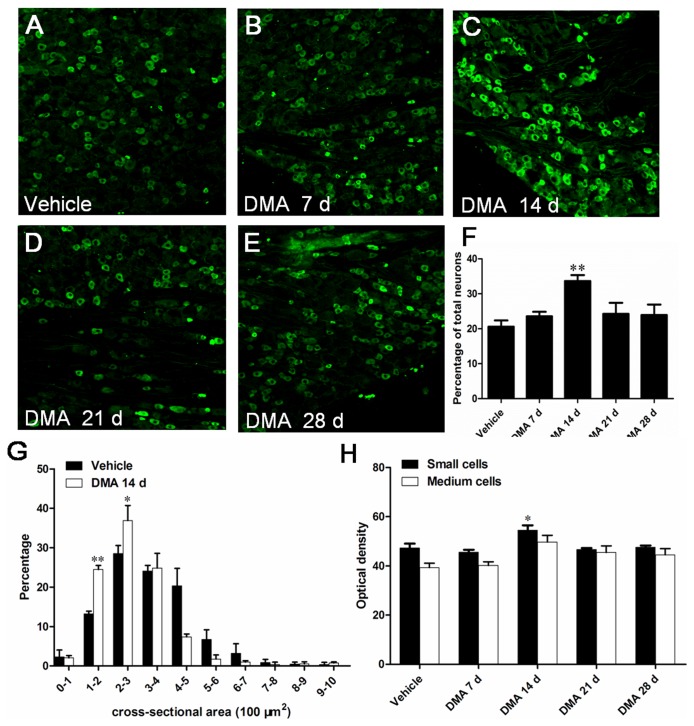
Immunofluorescent staining of TRPV1 and analysis of its expression pattern in DRG neurons at varying time points after the establishment of DMA. **A–E**, representative photographs of TRPV1 staining in DRG of different groups. **F**, illustration of the percentage of TRPV1-IR neurons over total neurons in vehicle, DMA 7 d, 14 d, 21 d and 28 d group (n = 4–6). **G**, histogram for size distribution of TRPV1-IR neurons. Note the decreased distribution of TRPV1 in neurons with cross-sectional area larger than 400–500 and increased distribution of this protein within the range of 100–400 µm^2^ (n = 4–6). **H**, comparison of the optical density due to TRPV1 immunostaining in small-sized (100–500 µm^2^) and medium-sized (500–1200 µm^2^) DRG neurons of different groups (n = 4–6). *P<0.05, **P<0.01 *vs*. vehicle control. Scale bar  = 100 µm.

To further corroborate the above observations, we next examined the distribution of the TRPV1-immunoreactive (IR) neurons in DRGs by immunohistochemical staining. TRPV1-IR neuron was clearly observed in all DRGs of rats (Fig. 3A–E). Consistent with the results of Western blot, the percentage of TRPV1-IR neurons over total neurons was notably higher on DMA 14 d, increasing from 20.6% (vehicle) to 33.7% (DMA 14 d; Fig. 3F, *P*<0.05). Size distribution analysis demonstrated that the cross-sectional area of 90.2% of TRPV1-IR neurons in vehicle group resided within the range of 100–500 µm^2^, while those in DMA 14 d group were more compacted in a small range of 100–400 µm^2^ (Fig. 3G). These observation suggest an increased TRPV1-IR population in small-sized DRG neurons during DMA progression. In addition, optical density (OD) analysis revealed that the OD of small-sized TRPV1-IR DRG (100–500 µm^2^) neurons was significantly increased in DMA 14 d group in comparison with vehicle or other experimental groups, whereas that of medium-sized TRPV1-IR neurons (500–1200 µm^2^) showed no statistically significant differences among all tested groups (Fig. 3H). These results together suggest that the increased TRPV1 protein expression observed by immunoblotting likely resulted from both the increased number of TRPV1-positive small-sized DRG neurons and increased TRPV1 protein expression therein.

**Figure 4:**
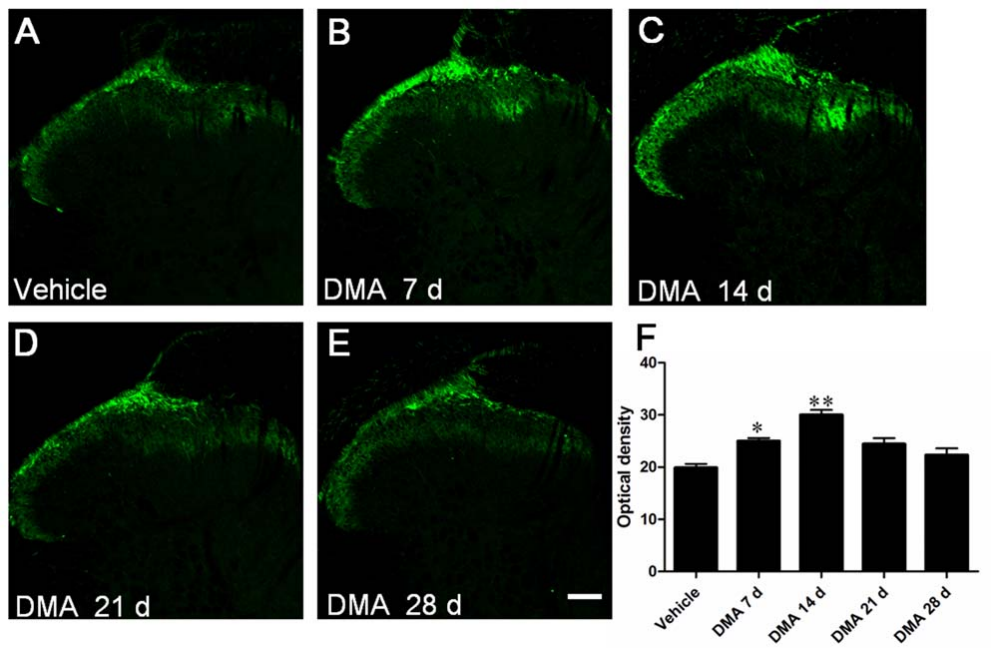
Immunofluorescent staining of TRPV1 in spinal dorsal horn (SDH) with the progression of DMA. **A–E**, representative photographs of TRPV1 staining in SDH in vehicle, DMA 7 d, 14 d, 21 d and 28 d groups. **F**, optical density analysis of TRPV1 staining in SDH showing the increased TRPV1-IR neurons on DMA 7 d and 14 d group (n = 4–6). Scale bar  = 100 µm.

**Figure 5:**
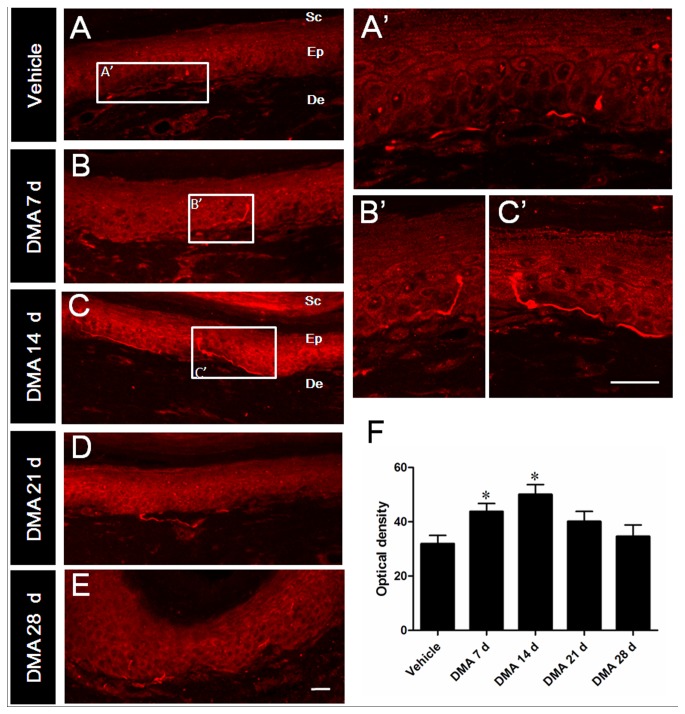
Immunofluorescent staining of TRPV1 in plantar skin of hind paw with the progression of DMA. A–E, typical photograsph of TRPV1 immunoreactivities in vehicle and DMA model rats at varied time points. A′–C′, the magnified pictures from the rectangle areas in A–C. F, optical density analysis of TRPV1 immunoreactivities in epidermis and dermis displaying strong enhancement on DMA 7 d and 14 d. Sc, Ep and De are the abbreviation of stratum corneum, epidermis and dermis, respectively. Scale bar  = 20 µm

In other series of experiments, we investigated TRPV1-immunoreactivity in central (SDHs; Fig. 4) and peripheral (plantar skin; Fig. 5) processes of DRG neurons. Consistent with previous reports, TRPV1 immunoreactivity was mainly located in the terminals of superficial dorsal horn of SC, especially in the laminae I and lamina II (Fig. 4A–E). As summarized in Fig. 4F, the OD of TRPV1-IR terminals in DMA 7 d and 14 d groups was remarkably higher than that of vehicle group. The density of TRPV1-IR products tended to decrease from DMA 21 d compared with DMA 14 d and finally returned almost completely to the vehicle level on DMA 28 d. TRPV1 distribution in plantar skins exhibited a similar temporal profile to that of SDHs. As shown in Fig. 5, TRPV1-IR nerve fibers and terminals were mainly distributed in the epidermis and superficial dermis. With the development of DMA, the density of TRPV1-IR fibers and terminals gradually increased to the peak from DMA 7 d to 14 d and then turned to decrease from 21 d to 28 d (Fig. 5A–E). These results collectively suggest that in the development of DMA, time-dependent increase of TRPV1 immunoreactivity in the central and peripheral terminals considerably coincides with that of DRG soma, although the latter exhibits a significant delay at the protein level (*i.e*. peaked on DMA 14 d; Fig. 2A and 3A–F).

### Neurochemical properties of TRPV1-IR DRG neurons

**Figure 6:**
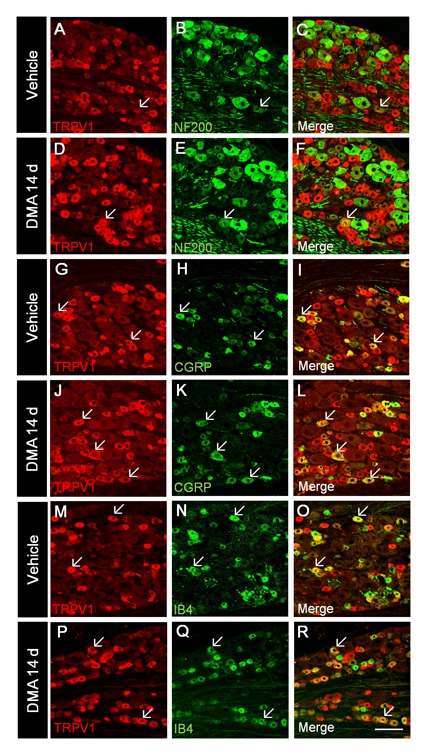
Double staining of TRPV1 with other neuronal markers in DRG. **A–R**, representative staining of TRPV1 (red) with NF200 (green; **A–F**), with CGRP (**G–L**), with IB4 (**M–R**) in vehicle-treated and DMA 14 d rats. Arrows indicate double-labeled neurons. Scale bar  = 100 µm.

DRG neurons can be classified into two types, *i.e*. those with and without myelinated fibers. NF200 can identify high molecular-weight neurofilaments enriched in large-diameter DRG neurons and their processes and thus is regarded as a marker for neurons with myelinated fibers [27]. Immunostaining of DRG with NF200 antibody revealed that during and after DMA, the percentage of NF200-IR over total neurons was significantly increased from 26.3% (vehicle) to 31.9% (DMA 14 d; Table 1). Nonetheless, a few percent of TRPV1/NF200 double-labeled neurons were observed in vehicle and DMA 14 d groups, which showed no statistically significant differences (indicated by arrows in Fig. 6A–F; the upper row of Table 2). To identify the neurochemical feature of increased TRPV1-IR neurons, CGRP and IB4, classical markers of peptidergic and nonpeptidergic DRG neurons, respectively [28], were employed for further immunostaining. The results showed that compared with vehicle group, the percentage of CGRP-IR to total DRG neurons was essentially increased in DMA 14 d group (Table 1). In support of this, the percentage of TRPV1/CGRP coincidence among CGRP-IR neurons was significantly increased in DMA 14 d group (Fig. 6G-L; Table 2, the middle row). By contrast, no obvious differences were observed in the percentage of TRPV1/IB4 dual-positive to IB4-IR neurons between the two groups under the same conditions (Fig. 6M-R; Table 2, the lower row). These findings strongly suggest that increased TRPV1-IR neurons in DRG pertain mainly to a class of CGRP-containing peptidergic neurons, but not to NF200-labeled or IB4-positive neurons, under DMA conditions.

**Table 1:** Percentage of NF200-, IB4- and CGRP-IR neurons in vehicle-treated and DMA 14 d groups (n = 4–6).

Markers	Percentage among total neurons	
	Vehicle group	DMA 14 d group
NF200	26.3 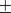 2.0	31.9 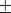 1.4**
CGRP	24.5 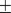 1.7	28.8 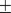 2.2*
IB4	34.9 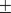 3.7	36.4 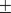 3.5

*P<0.05, **P<0.01 *vs*. vehicle group.

**Table 2:** Double labeling rate of TRPV1 immunoreactivity with that of NF200, CGRP or IB4 in DRG neurons (n = 4–6).

Markers	Vehicle group	DMA 14 d group
TRPV1+NF200/NF200	2.0 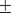 1.3	2.3 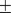 0.9
TRPV1+CGRP/CGRP	29.5 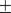 1.1	32.8 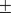 1.7*
TRPV1+IB4/IB4	71.6 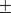 2.5	74.9 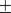 1.7

*P<0.05 *vs*. vehicle group.

### Effects of TRPV1 antagonists on DMA-related behaviors

**Figure 7:**
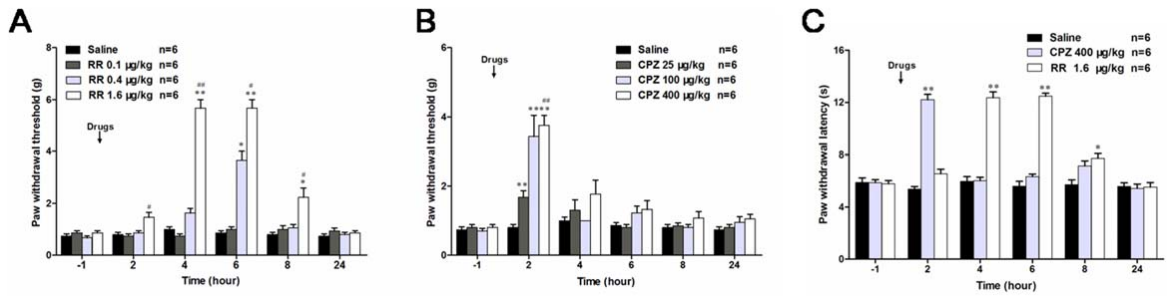
Single intrathecal application of TRPV1 antagonists alleviates mechanical allodynia and thermal hyperalgesia. **A** and **B**, single application of RR and CPZ at varing doses caused differential inhibition of mechanical sensitivity of DMA rats. **C**, single application of RR and CPZ at high dose caused significant inhibition of thermal hyperalgesia in DM rats. *P<0.05, **P<0.01 *vs*. vehicle control of the same time. ^#^ *P*<0.05, ^##^ *P*<0.01 *vs*. drugs of middle dose.

Finally, we investigated the effects of TRPV1 antagonists on DMA-related behaviors to evaluate the biological significance of increased TRPV1 expression after the establishment of DMA. In the first trial, single *i.t*. injection of RR or CPZ, the commonly used non-specific and specific inhibitors of TRPV1 respectively, was conducted in all tested animals. The results showed that *i.t*. administration of RR at a low dose (0.1 µg/kg) failed to induce any observable effects on animal behaviors. A medium dose of RR (0.4 µg/kg) significantly elevated PWT at 6 h, and a high dose of RR (1.6 µg/kg) advanced the onset of PWT elevation by 2 h, which was maintained for 4 hours (still significantly higher at 8 h after the injection; Fig. 7A). A single dose of CPZ (25, 100, 400 µg/kg, *i.t*.) markedly alleviated mechanical allodynia 2 h after its injection, but this effect quickly faded with time even at a high dose (400 µg/kg, Fig. 7B). Because TRPV1 plays a significant role in noxious heat transduction, we also tested the effects of RR and CPZ on DM-related thermal hyperalgesia. As shown in Fig. 7C, single injection (*i.t*.) of high-dose CPZ (400 µg/kg) or RR (1.6 µg/kg) prolonged the latency of paw withdrawal caused by noxious heat on DM 14 d with comparable potency. The effects of CPZ appeared earlier but lasted shorter than those of RR, as observed for DMA-related mechanical allodynia.

**Figure 8:**
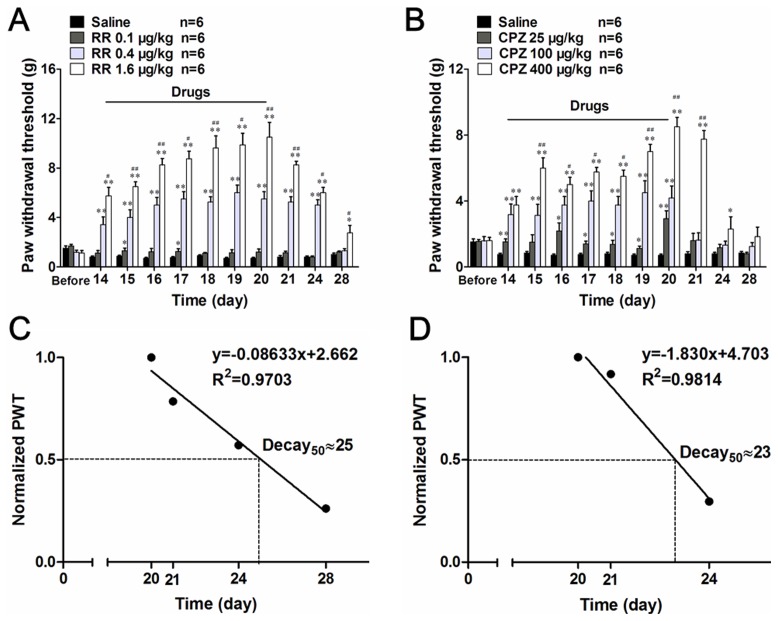
Multiple intrathecal applications of TRPV1 antagonists cumulatively antagonize mechanical allodynia. **A** and **B**, long-term effects of multiple applications of ruthenium red and capsazepine for consecutive 7 days (n = 6). **C** and **D**, reconstruction of the decay response diagram from **A** and **B** after the withdrawal of RR (**A**) and CPZ (**B**) (n = 6).The value in longitudinal axis was normalized by the thresholds value on DMA 20 d. The linear regressions were performed to calculate the decay rates and to evaluate the half decay times (‘Decay_50_’), and the results are shown above the plots. *P<0.05, **P<0.01 *vs*. vehicle control of the same time. ^#^ *P*<0.05, ^##^ *P*<0.01 *vs*. drugs of middle dose.

To further characterize the pharmacodynamic features of TRPV1 antagonists on DMA-related behaviors, we studied their cumulative effects by repeated administration from DMA 14 d to 20 d. Under these conditions, only the highest dose of RR (1.6 µg/kg) had discernible cumulative effects on PWT, where the maximal pain-relieving effect attained by the seventh administration (10.5
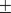
2.93; Fig. 8A) were twice larger than that produced by single administration (5.75
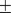
1.67; Fig. 7A). After the cessation of RR administration (DMA 21 d), the increased PWT slowly declined back to the control ‘sensitized’ level (DMA 28 d; Fig. 8A). We repeated the same protocol with CPZ to find that its highest dose (400 µg/kg) caused weaker cumulative effects on PWT than RR (9.25
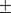
1.49; Fig. 8B). Regression analyses suggested that about 50% of the maximum effects on PWT still remained 5 days after withdrawal of RR, whereas this half decay time was significantly shorter for CPZ (3 days). These results suggest that RR may exert more potent and long-lasting antagonistic effects than CPZ on DMA-related behaviors (Fig. 8C and D).

## Discussion

In the present study, by applying a combination of molecular biological, morphological and behavioral pharmacological methods, we analyzed the spatio-temporal expression profile and functional implications of TRPV1 in STZ-induced rat DMA models. The results clearly demonstrated that; (1) in the soma of DRG neurons, expression of TRPV1 was significantly increased at both protein and mRNA levels between DMA 7 d and 14 d; (2) there was more preferential distribution of TRPV1 protein in the cellular processes of the DRG neurons; (3) the increased in TRPV1-IR neurons was rather confined to a small-sized, CGRP-IR subpopulation of primary afferent neurons; (4) *i.t*. administration of two TRPV1 antagonists, RR and CPZ, both effectively alleviated DMA-related behaviors, where the effects of RR were more potent and long-lasting than those of CPZ. Our present results revealed for the first time that the expression of TRPV1 was dynamically changed with the progression of DMA and suggest that the blockade of TRPV1 activity with RR or CPZ is an effective strategy not only to treat thermal hyperalgesia but also mechanical allodynia. These findings strongly suggest that TRPV1 plays an important role in nociceptive mechanosensing *in vivo*.

### Expression pattern of TRPV1 in DRG neurons with the development of DMA

In our study, the mRNA of TRPV1 in DRG was increased on DMA 7 d, while the up-regulation of TRPV1 protein was observed on DMA 14 d. Such a lag in protein expression suggests that enhanced transcription rather than facilitated trafficking/membrane insertion or reduced degradation may be responsible for DMA-related TRPV1 up-regulation. Notably, the up-regulation of TRPV1 protein in the processes and cell bodies of DRG neurons did not occur concurrently; the STZ-induced biphasic change (increase and decrease) of TRPV1 expression in the central and peripheral terminals preceded that in the soma (Fig. 2A *vs*. 2B). This seemingly paradoxical phenomenon was also supported by the results of immunostaining, which indicated that prominent changes of TRPV1-IR in DRG appeared on DMA 14 d, while those in SDH and skin, *i.e*. the central and peripheral processes of DRG neurons, emerged as early as DMA 7 d. It is well-known that proteins are initially synthesized in the soma and then transported to the terminals. Thus, changes of TRPV1 protein level in the soma (DRG neurons) should precede that of the terminal (SDH and skin). Nonetheless, a great deal of evidence suggest that TRPV1 acts as an important calcium source in the central and peripheral terminals of DRG neurons to facilitate the release of painful transmitters such as CGRP or SP which, on one hand, boosts the nociceptive signal transduction in the spinal cord, and on the other hand, exacerbates peripheral inflammation through so called neurogenic mechanisms [17], [29]. Therefore, we proposed that DRG neurons might distribute this essential molecule (TRPV1) in a ‘priority-of-use’ principle. In other word, TRPV1 protein may be preferentially transported to terminals after its synthesis rather than accumulated in the soma. This idea is analogous to the ‘activity-regulated protein synthesis’ in the hippocampal CA1 neurons, where dendrite-specific increase of a cytoskeleton associated protein Arc and its mRNA occurs without discernible changes in the soma [30]. Further studies with molecular engineering and high-resolution morphological techniques, which enabled the visualization of TRPV1 transport in DRG neurons *in vitro* and *in vivo*, will help to substantiate this hypothesis.

According to previous [31] and our present results, TRPV1 is mainly expressed in small- and medium-sized DRG neurons under normal conditions. Careful analysis of the distribution of TRPV1-IR DRG neurons with respect to DMA development revealed that increased TRPV1 expression occurred mainly in small-sized neurons ranging between 100–400 µm^2^. These results are consistent with the size-dependent redistribution of TRPV1-IR in DRG neurons induced by paclitaxel, a widely used anti-tumorigenic drug having the major side effects of painful paresthesia of the hands and feet [32]. Meanwhile, in an L_5_ and L_6_ spinal nerve ligation model, it is reported that small, strongly-stained TRPV1-IR neurons significantly decreased but medium/large, lightly-stained neurons increased ipsilaterally [33]. Differential patterns of TRPV1 expression in DRG neuronal populations may reflect complex neuronal phenotypic transformation under various pathophysiological conditions.

### Neurochemical features of increased TRPV1-IR neurons

Most nociceptive sensory neurons are divided into two classes by trophic support, *i.e*. nerve growth factor (NGF)-dependency and glial cell line-derived neurotrophic factor (GDNF)-sensitive [34]. CGRP and IB4 are recognized as the specific markers for NGF- and GDNF-sensitive neurons, respectively. In addition, with increasing recognition of the significance of Aβ fiber in pain processing, NF200 has become another important biochemical marker for neurons with myelinated fibers in DRG. Immunofluorescent staining of the three neuronal markers showed that the percentage of NF200-IR, CGRP-IR and IB4-binding neurons to total neurons in normal DRGs were consistent with those reported by previous studies [35]–[38]. After DMA formation, however, the numbers of CGRP-IR and NF200-IR neurons were significantly increased by about 20% (Table 1), indicating profound phenotypic transformation of DRG neurons under the persistent pain conditions. Partially consistent results were also obtained after sarcoma treatment [39], in which an increased population of CGRP-IR but not NF200-IR neurons was observed. The percentage of TRPV1-IR neurons in CGRP-IR neurons (but not NF200-IR or IB4-binding neurons) was significantly increased on DMA 14 d in our double immune-staining experiments (Table 2; from 29.5% to 32.8%; increasing by 11.2%). This implies that the DMA condition most prominently enhanced the expression of TRPV1 in a CGRP-ergic subpopulation of DRG neurons. It is reported that inflammatory hyperalgesia increased TRPV1 expression by about 31% in CGRP-positive subpopulations [40] but this did not occur when sarcoma injection was employed [39]. This discrepancy about TRPV1 residence in DRG neurons may reflect differential functional involvement of this channel in distinct pain models. TRPV1 can promote the release of neuropeptides (CGRP, SP or glutamate) from the afferent nerve terminals of DRG neurons under pain conditions [41]. We suppose that the functional relevance of TRPV1 to DMA development observed in the present study may also be mediated by facilitation of the release of CGRP. CGRP has long been regarded as a classic molecular marker of peptidergic nociceptive neurons, as mentioned above. However, whether CGRP-containing neurons actually function to sense certain noxious stimuli remains elusive. Ablation of CGRPα-containing neurons by toxin treatment has been reported to cause a profound loss of heat hypersensitivity in the complete Freund's adjuvant (CFA) model of inflammatory pain as well as and in the spared nerve injury (SNI) model of neurophathic pain, without affecting mechanical sensitivity/hypersensitivity [42]. This finding is in conflict with our present results, but the following two considerations may provide some account for it. Firstly, CGRP immunoreactivity reflects the expression of two akin peptides (CGRPα and CGRPβ) encoded by two separate genes (*Calca* and *Calcb*) in the DRG neurons [43]. Thus, the sole loss of CGRPα would not produce the whole dysfunctionality of CGRP-mediated signaling. Secondly, CGRPα expressing DRG neurons express other functional proteins. Thus, the consequences of ablating these neurons may not simply reflect the loss of CGRPα-mediated functions *in vivo*. More experiments, such as those that can discriminate the distribution of CGRPα and CGRPβ and elucidate their respective biological functions in DRG neurons will be needed to clearly answer these questions.

### Functional involvement of TRPV1 in DMA and the possible mechanism

RR and CPZ are the effective inhibitors of TRPV1 that act as a channel pore blocker and a competitive antagonist to against the TRPV1-selective agonist capsaicin, respectively [44]. In the present study, intrathecal application of RR and CPZ both effectively blocked DMA, but the effect was greater and more sustaining for the former than the latter, while appeared earlier for the latter. It is unclear why CPZ exerts such fast-onset and short-live analgesic effects, but similar actions of CPZ were previously reported for the inflammatory and neuropathic pain [11]. These results may be suggestive of the fast phamocodynamic features of CPZ. In heterologous expression system, both RR and CPZ dose dependently inhibited TRPV1-mediated Ca^2+^ response, but the antagonist affinities of CPZ was found lower than RR [45]. In fact, the analgesic efficacy of CPZ in rat is a matter of controversy. When *s.c*. injected, CPZ was shown to be ineffective to treat Freund's complete adjuvant induced inflammatory pain and partial ligation of the sciatic nerve induced neuropathic pain [11]. In contrast, the same dose of CPZ (also *s.c*.) was demonstrated to significantly inhibit paclitaxel induced thermal hyperalgesia in the rat. Such diversity mostly likely results from different pathological settings in distinct pain models employed by investigators, suggesting that TRPV1 may play differential roles in the peripheral signal processing depending on the types and modalities of pain.

RR, a water soluble polycationic dye, blocks the pore of TRPV1 and thus has been widely used as a detection tool for TRPV1 function both in *in vitro* and *in vivo* studies [11], [44]. However, RR is also known to nonspecifically inhibit several other TRP subtypes including TRPV2, TRPV3, TRPV4 and TRPA1 [46]. The results of the present study showed that RR was superior to a TRPV1-selective antagonist CPZ in the degree and duration of anti-allodynic actions with both single and multiple administrations. This immediately raises the possibility that part of the observed RR's effects may involve its non-specific actions on other mechanosensitive TRP channels than TRPV1, in particular, TRPV4 and TRPA1 [47]. In strong support of this speculation, we actually observed the dynamic changes of TRPV4 channel with similar temporal properties to TRPV1 in DMA rats (unpublished data). Thus, the molecular candidates for mechanical detection and transduction seem more complex than that for thermal detection and may involve the cooperation of TRPV1 with other TRP subtypes.

Despite highly plausible involvement of TRPV1 in mechanical allodynia or hyperalgesia [48] (and the present study), the mechanism underlying it is unclear so far. One possible explanation is, however, that some type of mechanical-biochemical conversion mechanism may operate therein [47]. Phospholipase A_2_ is a key component of major biochemical cascades of the cell that can be activated via various forms of mechanical stresses [49]. Once activated, PLA_2_ catalyzes the conversion of glycerophospholipids into free polyunsaturated fatty acids, such as arachidonic acid (AA) and lysophospholipids. AA is further catabolized to oxygenated products such as 12- hydroperoxyeicosatetraenoic acid (12-HPETE) which shares some degree of structural similarity with capsaicin and can act as an endogenous activator of TRPV1 [50]. It is thus possible that mechanical stresses activate neuronal TRPV1 channels via the PLA_2_-12-HPETE pathway to induce the mechanical hypersensitivity of afferent nerves. Consistent with this idea, recent studies reported that the expression level of PLA_2_ in DRG neurons was significantly elevated following compression injury or inflammation [51], [52]. Whether this pathway would contribute to the development of DMA *in vivo* will be an intriguing topic of the future study.

## Conclusions

The present study was designed to explore in a STZ-induced diabetes mellitus rat model whether the expression of TRPV1, a protein known to play an essential role in thermal hyperalgesia, is correlated with the development of mechanical allodynia. Our results clearly demonstrate that the expression of TRPV1 dynamically changes with the progression of DMA and that blockade of TRPV1 with RR or CPZ is an effective pharmacological intervention to antagonize both thermal hyperalgesia and mechanical allodynia. In conclusion, TRPV1 may play a central role in nociceptive mechanical signal processing *in vivo* and thus targeting TRPV1 may be of potential therapeutic significance to treat diabetic pains.

## Acknowledgments

We are grateful to Prof. Ryuji Inoue (Department of Physiology, School of Medical Sciences, Fukuoka University, Japan) for his critical comments and helpful language editing.
