# Correction: Spatio-Temporal Expression and Functional Involvement of Transient Receptor Potential Vanilloid 1 in Diabetic Mechanical Allodynia in Rats

**DOI:** 10.1371/journal.pone.0110164

**Published:** 2014-09-26

**Authors:** The PLOS ONE Staff

There is an error in the third sentence of the “Western blot” section of the Materials and Methods. The correct sentence is: The proteins were transferred onto polyvinylidene fluoride membrane (PVDF, φ = 0.45 µm, Millipore, Billerica, MA, USA).
